# Lysine-Specific Demethylase 1 (LSD1) Is Required for the Transcriptional Repression of the *Telomerase Reverse Transcriptase (hTERT)* Gene

**DOI:** 10.1371/journal.pone.0001446

**Published:** 2008-01-16

**Authors:** Qingjun Zhu, Cheng Liu, Zheng Ge, Xiaolei Fang, Xi Zhang, Klas Strååt, Magnus Björkholm, Dawei Xu

**Affiliations:** 1 Division of Hematology, Department of Medicine, Karolinska University Hospital Solna, Karolinska Institutet, Stockholm, Sweden; 2 College of Basic Medical Sciences, Shandong University of Traditional Chinese Medicine, Jinan, People's Republic of China; 3 Institute of Urology, The Second Hospital, Shandong University, Jinan, People's Republic of China; 4 Department of Obstetrics and Gynecology, Qilu Hospital, Shandong University, Jinan, People's Republic of China; Baylor College of Medicine, United States of America

## Abstract

**Background:**

Lysine-specific demethylase 1 (LSD1), catalysing demethylation of mono- and di-methylated histone H3-K4 or K9, exhibits diverse transcriptional activities by mediating chromatin reconfiguration. The *telomerase reverse transcriptase (hTERT)* gene, encoding an essential component for telomerase activity that is involved in cellular immortalization and transformation, is silent in most normal human cells while activated in up to 90% of human cancers. It remains to be defined how exactly the transcriptional activation of the *hTERT* gene occurs during the oncogenic process.

**Methodology/Principal Findings:**

In the present study, we determined the effect of LSD1 on hTERT transcription. In normal human fibroblasts with a tight *hTERT* repression, a pharmacological inhibition of LSD1 led to a weak hTERT expression, and a robust induction of hTERT mRNA was observed when LSD1 and histone deacetylases (HDACs) were both inhibited. Small interference RNA-mediated depletion of both LSD1 and CoREST, a co-repressor in HDAC-containing complexes, synergistically activated hTERT transcription. In cancer cells, inhibition of LSD1 activity or knocking-down of its expression led to significant increases in levels of hTERT mRNA and telomerase activity. Chromatin immunoprecipitation assay showed that LSD1 occupied the hTERT proximal promoter, and its depletion resulted in elevated di-methylation of histone H3-K4 accompanied by increased H3 acetylation locally in cancer cells. Moreover, during the differentiation of leukemic HL60 cells, the decreased hTERT expression was accompanied by the LSD1 recruitment to the hTERT promoter.

**Conclusions/Significance:**

LSD1 represses hTERT transcription via demethylating H3-K4 in normal and cancerous cells, and together with HDACs, participates in the establishment of a stable repression state of the *hTERT* gene in normal or differentiated malignant cells. The findings contribute to better understandings of hTERT/telomerase regulation, which may be implicated in the development of therapeutic strategies for telomerase dysregulation-associated human diseases including cancers.

## Introduction

The post-translational modification of nucleosomal histones, including acetylation, phosphorylation, methylation, ubiquitination and sumoylation, plays a key role in the regulation of gene transcription through remodelling chromatin structure [1]. Among all above modifications, regulated methylation of six known lysine (K) residues within the tails of histone H3 (H3-K4, H3-K9, H3-K27, H3-K36 and H3-K79) and H4 (H4-K20) is unique as mono- (M1), di- (M2) and tri- (M3) three modification states can be generated [2], [3]. These different states, together with the identity of the modified lysine residues, result in diverse consequences on transcriptional activities. Methylation of H3-K4, H3-K36 and H3-79 is predominantly associated with transcriptionally active genes, whereas methylation of H3-K9, H3-K27 and H4-K20 marks silent genes or heterochromatin [1]–[3].

It is well known that histone acetylation and phosphorylation are dynamic and reversible, while histone methylation has long been thought as a static modification [1]–[3]. However, the recent discovery of multiple histone demethylases (HDMs) has totally changed this dogma, and thus the dynamic histone H3 methylation–mediated transcriptional regulation has emerged as a subject of intense investigations [2]–[5]. Lysine-specific demethylase 1 (LSD1), the first identified HDM [6], has attracted a great deal of interest because of its broad functional activities in transcriptional programs [7]–[15]. By interacting with diverse co-factors and catalyzing demethylation of mono- and di-methylated H3-K4 or K9, LSD1 is capable of either repressing or activating the target genes. For instance, when recruited to the target promoters by the androgen receptor, LSD1 removed the repressive H3-K9(M2) mark, thereby driving the androgen target activation [7], [11]–[13]. On the other hand, LSD1 specifically demethylates H3-K4(M1) and (M2) that in turn leads to gene repression by maintaining a unmethylated H3-K4 status on its target promoters [6], [8], [13], [16], [17]. By eliciting such dual effects, LSD1 has been implicated in embryonic development, cell differentiation and proliferation, stem and cancer cell biology.

Telomerase, a RNA-dependent DNA polymerase silent in most normal human somatic cells, is frequently activated during the oncogenic process [18], [19]. The compelling evidence suggests that activation of telomerase is essential for cellular immortalization and malignant transformation [18], [19]. Moreover, telomerase and/or its components have also been implicated in the regulation of stem cell mobilization and proliferation [20], [21]. It has been established that the stable repression of the telomerase catalytic subunit, encoded by the *telomerase reverse transcriptase (hTERT)* gene, is a predominant event resulting in the lack of telomerase activity in most normal cells [22]–[26]. Therefore, in order to acquire telomerase activity, it is necessary to erase negative regulators suppressing hTERT transcription. However, it remains incompletely understood how the repression or activation of the *hTERT* gene in normal and cancerous cells is achieved, despite great efforts in elucidating the underlying mechanisms during the past years.

Recent studies have started to uncover a close association between histone modifications including methylation and transcriptional activity of the *hTERT* gene in human cells [27]–[35]. Lack of histone H3-K4 methylation generally marks the repressive hTERT transcription in telomerase-deficient cells while the over-expression of SMYD3, a histone methyltransferase (HMT) that specifically di- and tri-methylates histone H3-K4, leads to the induction of hTERT mRNA expression via enhancing methylated H3-K4 at the hTERT proximal promoter region [34], [35]. It has been shown that the transcription factor c-MYC, a key activator of the *hTERT* gene [36], [37], is capable of binding to its target promoters only when the H3-K4 is methylated within appropriate regions of target promoters [38]. Conceivably, histone H3-K4 demethylation at the hTERT promoter may be required to lock the chromatin in a closed state in telomerase-deficient cells. However, it remains unknown which HDMs are associated with the hTERT promoter and responsible for maintaining H3-K4 demethylation locally. In the present study, we sought to address this issue by determining the potential role for LSD1 in controlling hTERT transcription.

## Results

### Tranylcypromine treatment or depletion of LSD1 expression induces hTERT mRNA expression in MRC5 fibroblasts

**Figure 1:**
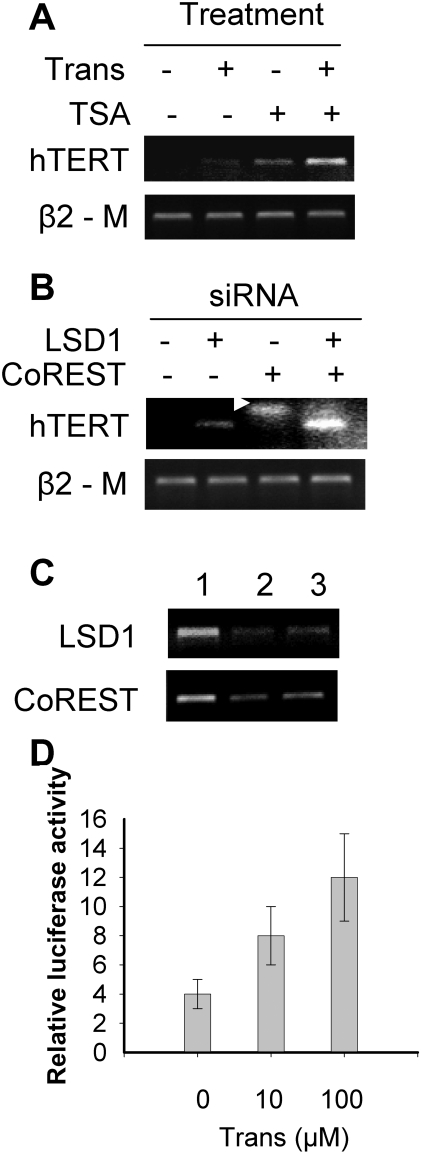
The effect of LSD1 inhibition on the hTERT mRNA expression or gene transcription in human normal fibroblast MRC5 cells. (A) The induction and synergistic up-regulation of hTERT mRNA expression in MRC5 cells by the LSD1 inhibitor tranylcypromine alone and its combined treatment with the histone deacetylase (HDAC) inhibitor TSA, respectively. The cells were incubated with either tranylcypromine at 25.0 µM or TSA 0.1 µM, or both of them overnight and then harvested for hTERT mRNA analyses using RT-PCR. Trans: tranylcypromine. (B) The synergistic effect of LSD1 and CoREST depletion on the induction of hTERT mRNA in MRC5 cells. The knocking-down of LSD1 and CoREST expression was performed using the specific siRNA targeting LSD1 and CoREST. Arrow: primer dimmers. (C) The demonstration of efficient down-regulation of the target genes in siRNA-transfected cells. Lane 1: Control siRNA; lane 2: LSD1 (Upper) and CoREST siRNAs (Lower), respectively; and lane 3: LSD1+CoREST siRNAs. Note the results in (B) and (C) were from the same set of cDNA, and therefore, β2 –M was only shown in (B). (D) The up-regulation of the hTERT promoter activity by tranylcypromine. The p181 reporter construct that harbors the hTERT proximal promoter sequence was transfected into MRC5 cells in the absence or presence of tranylcypromine, and then analysed for luciferase activity 48 hours later. Variation in transfection efficiency was normalized by the TK-driven *Renilla* luciferase activity. Columns: Relative luciferase activity arbitrarily expressed as the ratio of p181/TK; Bars: SD.

The amino oxidase inhibitor tranylcypromine has been identified to potently suppress the enzymatic activity of LSD1 [17]. Therefore, to elucidate a potential role for LSD1 in the transcriptional regulation of the *hTERT* gene, we first incubated normal human MRC5 fibroblasts exhibiting a stable *hTERT* repression in the presence of tranylcypromine. As shown in Fig. 1A, hTERT transcripts were not observed in control MRC5 cells while tranylcypromine treatment induced a weak but detectable expression of hTERT mRNA. To rule out possible LSD1-independent, non-specific effects of tranylcypromine, we knocked-down LSD1 expression by using the specific LSD1 siRNA. siRNA treatment of MRC5 cells resulted in silencing of LSD1 expression, and consistent with the finding described above, hTERT mRNA was induced in these LSD1-depleted cells (Fig. 1B). Taken together, both pharmacological inhibition of LSD1 activity and depletion of its expression are capable of de-repressing the hTERT transcription in normal human fibroblasts, although the effect was not robust.

### The simultaneous inhibition of both LSD1 and HDACs is required for the optimal induction of hTERT mRNA expression in MRC5 cells

It has been previously shown that the inhibition of HDAC activity by TSA activates hTERT transcription in human fibroblasts and other cell types [27], [28], [31]–[33], [39]. Moreover, an intimate interplay between LSD1 and HDACs has been observed [40]. We thus sought to probe whether a synergistic induction of hTERT expression could be achieved by inhibiting both LSD1 and HDAC activities. For this purpose, MRC5 cells were exposed to either tranylcypromine or TSA alone, or tranylcypromine plus TSA. A weak expression of hTERT mRNA was observed in cells treated with tranylcypromine at 25 µM or TSA at 0.1 µM, whereas the cell exposure to both tranylcypromine and TSA led to a robust increase in hTERT transcripts (Fig. 1A). The data suggest that LSD1 and HDACs cooperate to maintain a repressive state of the *hTERT* transcription in normal human MRC5 cells.

It is known that LSD1 interacts with CoREST, a co-repressor that exists in the HDAC-containing complex [8], [16]. To corroborate the specific effect of tranylcypromine and TSA, we thus depleted both LSD1 and CoREST expression in MRC5 cells using a siRNA approach. The efficient knocking-down of these transcripts was verified with use of RT-PCR (Fig. 1C). The transfection of LSD1 but not CoREST siRNA into MRC5 cells induced minimal amounts of hTERT mRNA whereas the depletion of both of them synergistically activated hTERT transcription (Fig. 1B), which was in good accordance with the results achieved in MRC5 cells incubated with both tranylcypromine and TSA.

### The hTERT promoter is activated in MRC5 cells in the presence of tranylcypromine

Next we sought to examine the effect of tranylcypromine on the hTERT promoter activity. p181, a reporter construct with an insert of the core hTERT promoter sequence [37], was transfected into MRC5 cells and luciferase activity driven by the hTERT core promoter was assessed 48 hours post-transfection in the presence or absence of tranylcypromine. As expected, the p181 activity in untreated MRC5 cells was very low, consistent with earlier observations. The exposure of the cells to tranylcypromine substantially increased the hTERT promoter activity in a dose-dependent manner (Control vs tranylcypromine treatment at 10 and 100 µM, p<0.05, paired Wilcoxon two sample test) (Fig. 1D).

### Tranylcypromine up-regulates hTERT expression and telomerase activity concomitant with elevated H3-K4(M2) and H3 acetylation at the hTERT proximal promoter in cancer cells

**Figure 2:**
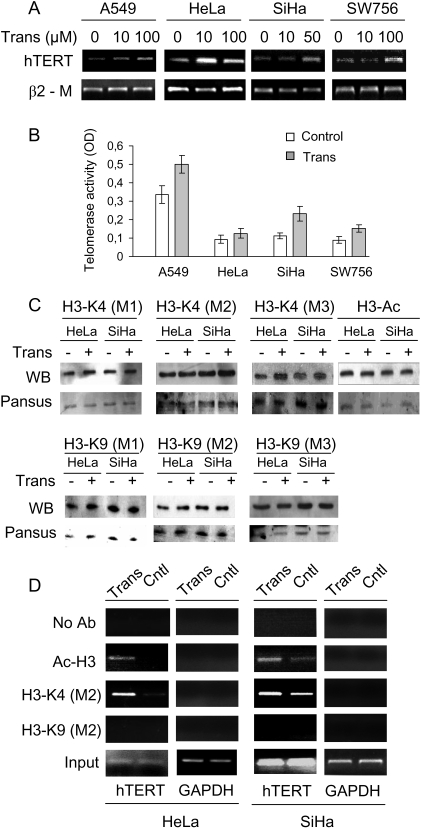
The tranylcypromine-induced up-regulation of hTERT mRNA expression and telomerase activity accompanied by increase in H3-K4(M2) and H3 acetylation at the hTERT proximal promoter region in human cancer cells. (A) The dose-dependent up-regulation of hTERT mRNA expression in A549, HeLa, SiHa and SW756 cells treated with tranylcypromine. (B) Telomerase activity in the same sets of cells treated with tranylcypromine (Trans) at 100 µM (for A549, HeLa and SW756) or 50 µM (for SiHa) as in (A). Telomerase activity was assessed using a telomerase PCR ELISA kit and arbitrarily expressed as absorbance (OD450–OD690). Columns: Relative telomerase activity; Bars: SD. (C) The western blot analysis of global histone H3 acetylation, H3-K4, and H3-K9 mono-, di- and tri-methylation in HeLa and SiHa cells treated with tranylcypromine (Trans). Pansus staining was shown for equal histone protein loads. (D) The increased histone H3-K4(M2) and H3 acetylation at the hTERT proximal promoter in tranylcypromine-treated HeLa and SiHa cells. Representative ChIP results were shown.

Having demonstrated an inhibitory role for LSD1 in the hTERT transcription in human normal fibroblasts, we wanted to further clarify its regulatory effect on hTERT expression in different cancer cells. The cervical cancer lines HeLa, SiHa and SW756, and a lung cancer line A549 cells were incubated with different concentrations of tranylcypromine overnight and were then analyzed for changes in hTERT mRNA and telomerase activity. The results documented in Fig. 2A showed a significant up-regulation of hTERT mRNA expression in all tranylcypromine-treated cells although the sensitivity varied from cell line to cell line. HeLa cells were most sensitive to tranylcypromine treatment and the concentration at 10 µM induced optimal increases in hTERT mRNA. Consistently, an increase in telomerase activity was observed in the same sets of A549, SiHa and SW756 cells as well (Fig. 2B). HeLa cells treated with 100 µM tranylcypromine exhibited slight enhancement in telomerase activity, in accordance with hTERT mRNA results.

Given tranylcypromine's inhibitory effect on LSD1 activity [17], we next asked whether potential alterations in histone H3 methylation occurred in cells treated with tranylcypromine. We first recorded a change in global histone H3 methylation and acetylation profile in control and treated cells, but failed to find a significant difference in total methylated H3-K4 and K9 or H3 acetylation (Fig. 2C). Chromatin immunoprecipitation (ChIP) assays were then applied to determine histone H3 modifications at the hTERT promoter region by using the PCR primers spanning the proximal promoter regions (between –296 and –22 relative to ATG). The treatment of HeLa cells with the LSD1 inhibitor led to substantial enrichments in H3-K4(M2) at the hTERT core promoter region (Fig. 2D). Similar alterations were observed in SiHa cells following tranylcypromine treatment: H3-K4(M2) at the hTERT promoter was increasingly enriched. Furthermore, highly increased histone H3 acetylation at the hTERT promoter region was found in both HeLa and SiHa cells in the presence of tranylcypromine (Fig. 2D). In contrast, no significant H3-K9 methylation(M2) was detected at the hTERT proximal promoter (Fig. 2D). These data indicate that LSD1 specifically demethylates H3-K4 rather than K9 associated with the hTERT proximal promoter, consistent with its inhibitory effect on the hTERT transcription. Additionally, we found that the altered H3-K4 modification was limited to the core promoter region and did not extend to the promoter nucleosomes further upstream (>−700 and beyond; data not shown).

### Depleting LSD1 expression induces higher levels of hTERT mRNA and telomerase activity via inhibiting H3-K4 demethylation at the hTERT proximal promoter in cervical cancer cells

**Figure 3:**
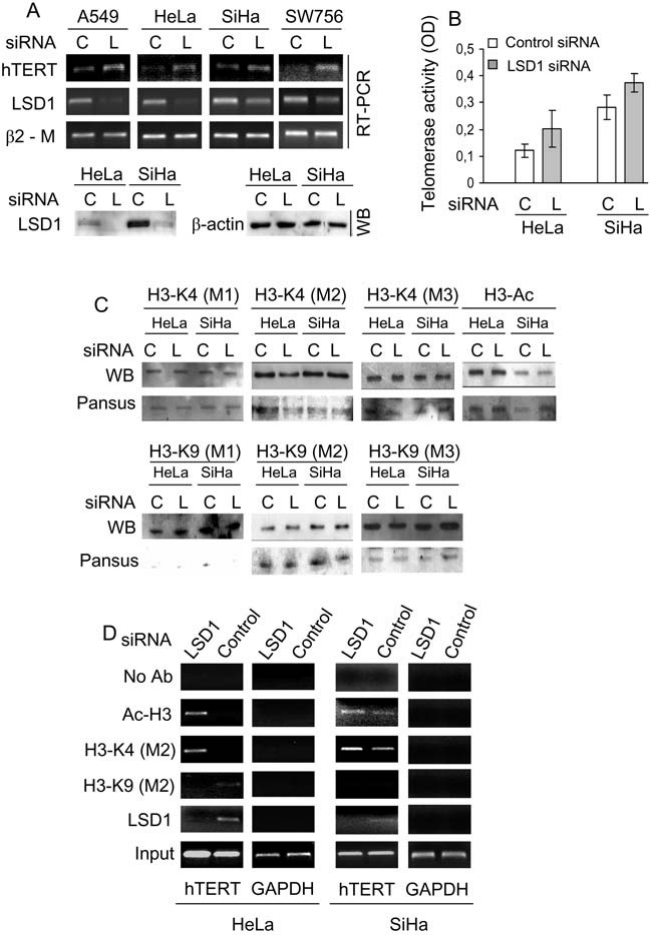
LSD1-depletion mediated up-regulation of hTERT and telomerase expression through enhancing H3-K4(M2) and H3 acetylation at the hTERT proximal promoter in cancer cells. (A) The up-regulation of hTERT mRNA expression induced by depletion of LSD1 in human cancer cells. RT-PCR for LSD1 mRNA was shown to verify efficient depletion of LSD1 expression in those cells. The lower panel further demonstrated an inhibition of LSD1 expression at protein levels in HeLa and SiHa cells treated with LSD1 siRNA. WB: Western blot. (B) The enhanced telomerase activity in HeLa and SiHa cells with LSD1 depletion. (C) The western blot analysis of global histone H3 acetylation, H3-K4, and H3-K9 mono-, di- and tri-methylation in HeLa and SiHa cells transfected with LSD1 specific siRNA. Pansus staining was shown for equal histone protein loads. (D) The abolishment of LSD1 concomitant with the increased H3-K4(M2) and H3 acetylation at the hTERT proximal promoter in LSD1-depleted HeLa and SiHa cells. Representative ChIP results were shown. C and L: Control and LSD1 siRNA, respectively.

To obtain independent evidence for the inhibitory effect of LSD1 on hTERT transcription and telomerase activity, LSD1 expression was further knocked-down in HeLa and SiHa cells again using a siRNA strategy. As shown in Fig. 3A, LSD1 could be efficiently depleted in HeLa and SiHa cells. The up-regulation of hTERT mRNA and telomerase activity was observed in both cells, consistent with those documented in tranylcypromine-treated cells (Fig. 3A and B).

The depletion of LSD1 in HeLa and SiHa cells did not lead to global changes in the histone H3 methylation and acetylation profile (Fig. 3C). We then performed ChIP analyses to define potential alterations in H3 methylation associated with the hTERT proximal promoter in LSD1-depleted cells. The presence of LSD1 on the hTERT promoter was readily observed in the control cells while it became undetectable following LSD1-specific siRNA treatment (Fig. 3D). The depletion of LSD1 expression was accompanied by significant increases in H3-K4(M2) and histone H3 acetylation at the hTERT promoter in both HeLa and SiHa cells (Fig. 3D). There were no detectable histone H3-K9(M2) at the hTERT promoter region in either control or LSD1-depleted cells (Fig. 3D). These results provide evidence that LSD1 targets the *hTERT* gene and represses hTERT transcription via specifically demethylating histone H3-K4 (M2) at the hTERT proximal promoter in cervical cancer cell lines.

### The recruitment of LSD1 to the hTERT promoter accompanied by a loss of H3-K4(M2) and down-regulation of hTERT expression occur concomitantly during the terminal differentiation of leukemic HL60 cells

**Figure 4:**
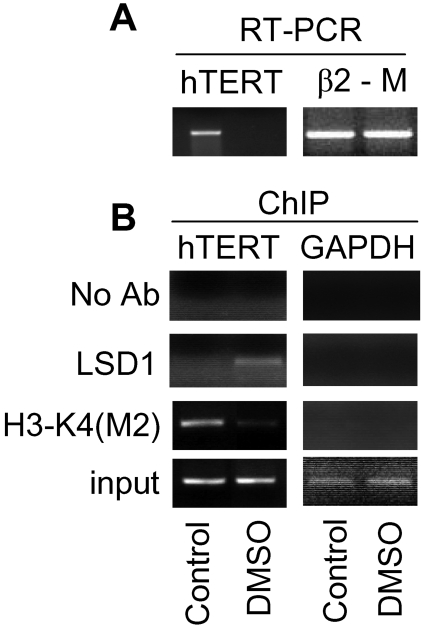
The recruitment of LSD1 to the hTERT proximal promoter concomitant with repression of hTERT transcription in the differentiated HL60 cells. (A) The down-regulation of hTERT mRNA expression in HL60 cells undergoing terminal differentiation induced by DMSO treatment. (B) The presence of LSD1 concomitant with a loss of H3-K4(M2) at the hTERT proximal promoter region in differentiated but not undifferentiated HL60 cells. Control: Undifferentiated HL60 cells and DMSO: DMSO-induced differentiated HL60 cells.

It has been well clarified that HL60 leukemic cells, when induced to undergo terminal differentiation, exhibit a stable repression of hTERT transcription [39], [41]–[45]. To explore whether LSD1 is involved in the establishment of such a repressive state of the *hTERT* gene in the differentiated HL60 cells, we treated the cells with DMSO, a differentiation inducer, and then analysed binding of LSD1 to the hTERT proximal promoter. As expected, the DMSO exposure of HL60 cells triggered complete cessation of hTERT transcription within 48 hours (Fig. 4A). The ChIP results showed that LSD1 recruitment to the hTERT promoter did not occur in undifferentiated cells while it was readily observed in differentiated cells treated with DMSO for 48 hours, concomitant with the loss of H3-K4(M2) at the hTERT proximal promoter (Fig. 4B).

## Discussion

The methylated histone H3-K4 and K9 mark gene activation and repression, respectively [1]–[3]. LSD1, the first identified histone demethylase [6], has been shown to either repress a cohort of target genes through demethylation of H3-K4(M2) or activate other targets via removing the repressive mark H3-K9(M2), dependent on its interaction with different partners [6]–[8], [12], [17]. In the present study, we provide evidence that LSD1 is required for transcriptional repression of the *hTERT* gene in both normal and cancerous human cell lines.

Previous observations showed that the entire *hTERT* gene was embedded in a highly condensed chromatin in human normal fibroblasts with a tight repression of hTERT transcription [29], [30]. When treated with a HDAC inhibitor, the local chromatin became open due to the increased histone acetylation, which thus allowed the transcriptional activation of the *hTERT* gene [22]–[26]. These data indicate an important role for HDAC-mediated histone deacetylation in repressing *hTERT* expression in telomerase-deficient cells. It is evident from the present investigation that LSD1 is similarly required for the establishment of repressive hTERT chromatin in normal fibroblasts. The activation of an optimal hTERT transcription can be achieved only when LSD1 and HDACs are both inhibited. Taken together, LSD1-mediated H3-K4 demethylation and HDAC-mediated histone deacetylation cooperate to establish a repressive transcriptional environment at the hTERT promoter region in human normal fibroblasts. Moreover, the recruitment of LSD1 to the hTERT promoter observed in differentiated HL60 cells provides independent evidence for its participation in the stable repression of the *hTERT* gene. It is currently unclear whether other histone demethylases are involved in the H3-K4 demethylation at the hTERT chromatin. This issue is now being addressed in our laboratory.

Intriguingly, the temporal patterns of expression of specific components of the LSD1 complex during the mammalian development cause a switch of LSD1 function from repression to activation, or reverse [13]. Therefore, we compared the transcriptional effect of LSD1 on the *hTERT* gene between cancer and normal cells. All the tested cancer cells including HeLa, SiHa, SW756 and A549 expressed hTERT mRNA and telomerase activity, but the pharmacological inhibition of LSD1 activity or depletion of its expression was capable of further up-regulating hTERT mRNA and telomerase activity levels, which clearly indicates an identical inhibitory effect of LSD1 on hTERT transcription in both normal and cancer cells. More importantly, the ChIP result demonstrated the LSD1 occupancy on the hTERT proximal promoter. Consistent with its functional activity, inhibition of LSD1 led to increased histone H3-K4 (M2) at the hTERT promoter region. In contrast, there were no detectable changes in H3-K9 methylation locally. It seems that LSD1 only targets H3-K4 for its demethylation at the hTERT promoter. Moreover, the LSD1-mediated H3-K4 demethylation was confined to the core promoter region without a further extension to upstream nucleosomes based on our unpublished data.

H3-K4 methylation alters the chromatin folding, leading to increased accessibility of DNA to proteins that mediate transcription [1]–[3]. It has been shown that the methylated H3-K4 is one of the prerequisites for the E-box binding by c-MYC on MYC target promoters [34], [38]. Given the fact that *hTERT* gene is a direct target of c-MYC [36], [37], the LSD1-mediated H3-K4 demethylation might prevent the c-MYC binding to the hTERT promoter, which in turn attenuates a transcriptional activity of the *hTERT* gene. Consistent with this hypothesis, we indeed observed that the depletion of LSD1 led to not only increased H3-K4(M2) but also H3-K4(M3) at the hTERT proximal promoter in HeLa cells (data not shown). In addition, recent studies have revealed an intimate cross-talk between LSD1 and deacetylating enzymes, and histone demethylation can be a secondary target of HDAC inhibitors [40]. LSD1 interacts with CoREST which is in the HDAC-containing complex [8], [16], and thus it may further repress gene transcription program via indirectly promoting histone deacetylation. Consistently, we observed concomitant increases in histone H3-K4(M2) and H3 acetylation in cervical cancer cells treated with either LSD1 inhibitor or its specific siRNA. Taken together, LSD1 is capable of regulating the chromatin structure of the hTERT promoter region at multiple levels.

Although LSD1 was observed to occupy the hTERT proximal promoter region, as demonstrated in our ChIP assay, it remains unclear how it is recruited there. We examined a potential interaction of LSD1 with the transcription factors Sp1 and MYC family members, the key regulators for hTERT transcription, and none of them were associated with LSD1 under our immunoprecipitation settings. It is thus plausible that LSD1 physically associates with other co-factors that are in turn tethered to the hTERT proximal promoter.

To conclude, the present study demonstrates that the histone demethylase LSD1 is required for the transcriptional repression of the *hTERT* gene in both normal and cancerous cells. By directly demethylating H3-K4 and indirectly promoting H3 acetylation associated with the hTERT core promoter, LSD1 participates in the establishment of a stable repression state of the *hTERT* gene in human normal or differentiated malignant cells. Our findings thus provide insights into the regulatory mechanism underlying telomerase silencing and activation in human cells, and have implications in cancer and stem cell biology.

## Materials and Methods

### Cell lines, culture conditions, and chemicals

Human cervical cancer cell lines HeLa, SiHa and SW756, lung carcinoma cell line A549, normal fetal lung fibroblasts MRC5, and leukemic cell line HL60 were cultured at 37°C/95%air/5%CO_2_ in RPMI 1640 medium (Life Technologies, Paisley, Scotland, UK) containing 10% fetal calf serum, 100 units/ml penicillin, and 2 mM L-glutamine. The specific LSD1 inhibitor tranylcypromine and histone deacetylase (HDAC) inhibitor trichostatin A (TSA) were purchased from Biomol International LP (Plymouth Meeting, PA, USA) and Sigma-Aldrich Sweden AB (Stockholm, Sweden), respectively. HL60 cells were treated with 1.25% DMSO for 48 hours to induce their terminal differentiation [39].

### siRNA treatment

Chemical modified Stealth™ siRNA targeting LSD1 and CoREST, and control siRNA were bought from Invitrogen (Carlsbad, CA, USA). The sequences for LSD1 and CoREST were UUU CCA UGA UAC CAG CAG CUU CUC C and AAG AUU GUC CCG UUC UUG ACU GCG U, respectively. MRC5 and cervical cancer cells were transfected with siRNA using Lipofectamine2000 (Invitrogen) according to the manufacturer's protocol.

### Total RNA extraction and RT-PCR

Total cellular RNA was extracted using ULTRASPEC™-II RNA kit (Biotecx Lab, Houston, TX, USA). cDNA synthesis, the RT-PCR primers and conditions for *hTERT* mRNA were as described previously [46]. β2-M mRNA expression was used as a control for RNA loading and RT efficiency and was amplified with its specific primers for 26 cycles. PCR for both hTERT and β2-M mRNA was optimised to keep amplification in a linear phase, which allowed a semi-quantitative evaluation for the level of hTERT transcript as described [46]. The PCR primers for LSD1 mRNA are 5′-GAC TTC TTG GCA GAG TTG TC-3′ (forward) and 5′-GTG AAA GAG TTG CAG ATC C-3′ (reverse). RT-PCR for CoREST mRNA was carried out using the following primer pair: (forward) 5′-GGG ATG CTC TTC TGG CAT AA-3′ and (reverse) 5′-GGA GGT TTC CTT TTT GCT CTA-3′.

### Histone and total cellular protein extraction, and western blot

Preparation of a total histone fraction from nuclei was done by extraction with a dilute acid as described [33]. Total cellular proteins were extracted with RAIP lysis buffer. Two µg of histone proteins or 20 µg of total cellular proteins were resolved by sodium dodecyl sulate-polyacrylamide gel electrophoresis and transferred to an intracellulos membrane. The membranes were probed with the specific antibodies against methylated histone H3-K4(M1, M2 and M3), H3-K9(M1, M2 and M3), acetylated H3 or LSD1 (Upstate, NY, USA) followed by anti-mouse horseradish peroxidase-conjugated IgG and developed with the enhanced chemiluminescent method (ECL, Amersham, UK).

### Telomerase activity assay

TeloTAGGG Telomerase PCR ELISA (Roche Diagnostics Scandinavia AB, Stockholm, Sweden) based on Telomeric Repeats Amplification Protocol was used to determine telomerase activity in all samples in duplicate according to manufacturer's instruction (40). One µg of protein from total cell lysates was added into the reaction mixture and the generated telomere product was PCR-amplified using 23 cycles. Four µl of products was used for ELISA assay and the level of telomerase activity was arbitrarily expressed as absorbance (OD450–OD690).

### Luciferase activity assay

Transfection for luciferase activity assay was performed in 24-well plates using Lipofectamine2000 (Invitrogen). The hTERT luciferase reporter (p181), kindly provided by Dr. S Kyo (Kanazawa University, Japan), harbors the core promoter sequence of the hTERT 5′-flanking region [37]. p181 was co-transfected with a Renilla reniformis luciferase-containing plasmid, which is under control of the thymidine kinase (TK) promoter. The transfected cells were then treated with different concentrations of tranylcypromine. Luciferase activity in the cell lysates was determined by using a dual luciferase reporter assay system (Promega, WI, USA) 48 hrs post-transfection, and the hTERT promoter-driven firefly luciferase activity was normalized to the TK renilla activity.

### ChIP assay

ChIP assay was done as described [33], [34]. The HeLa, SiHa and HL60 cells, under different treatment conditions, were cross-linked by incubating them in 1% (vol/vol) formaldehyde-containing medium for 10 mins at 37°C and then sonicated to make soluble chromatin with DNA fragments between 200 and 1000 bps. The antibodies against LSD1, H3-K4(M2), H3-K9(M2), and acetylated histone H3 (Upstate, NY, USA) were used to precipitate DNA fragments bound by their corresponding elements. The protein-DNA complex was collected with protein A or G Sepharose beads (Upstate), eluted, and reverse cross-linked. Following treatment with Protease K (Sigma), the samples were extracted with phenol-chloroform and precipitated with ethanol. The recovered DNA was re-suspended in TE buffer and used for the PCR amplification as described [33], [34]. The PCR primers for the proximal promoter regions (spanning between –296 and –22 relative to ATG) were 5′-CCA GGC CGG GCT CCC AGT GGA T-3′ (forward) and 5′-GGC TTC CCA CGT GCG CAG CAG GA-3′ (reverse). The primers for GAPDH were described elsewhere [33].
